# Path modeling of knowledge, attitude and practice toward palliative care consultation service among Taiwanese nursing staff: a cross-sectional study

**DOI:** 10.1186/s12904-017-0228-6

**Published:** 2017-08-17

**Authors:** Hsueh-Hsing Pan, Hsiu-Ling Shih, Li-Fen Wu, Yu-Chun Hung, Chi-Ming Chu, Kwua-Yun Wang

**Affiliations:** 1 School of Nursing, National Defense Medical Center, No.161, Sec. 6, Minquan E. Rd., Neihu Dist., Taipei City, 114 Taiwan; 2 Department of Nursing, Tri-Service General Hospital, Taipei City, Taiwan; 3 School of Public Health, National Defense Medical Center, Taipei City, Taiwan; 4 Department of Nursing, Taipei Veterans General Hospital, Taipei City, Taiwan; 5 School of Nursing, Yang-Ming University, Taipei, Taiwan

**Keywords:** Palliative care consultation service, Knowledge, Attitude, Practice, Path modeling

## Abstract

**Background:**

The Taiwanese government has promoted palliative care consultation services (PCCS) to support terminally ill patients in acute ward settings to receive palliative care since 2005. Such an intervention can enhance the quality of life and dignity of terminally ill patients. However, research focusing on the relationship between the knowledge, attitude and practice of a PCCS using path modelling in nursing staff is limited. Therefore, the aim of this study was to elucidate the effect of path modeling on the knowledge, attitude and practice toward PCCS in Taiwanese nursing staff.

**Methods:**

This was a cross-sectional, descriptive study design using convenience sampling. Data collected included demographics, knowledge, attitude and practice as measured by the PCCS inventory (KAP-PCCSI). Two hundred and eighty-four nursing staff from a medical center in northern Taiwan participated in the study in 2013. We performed descriptive statistics, regression analysis, and path modeling using SPSS 19.0 and set *p* < 0.05 as the statistical significance threshold.

**Results:**

The results showed that the identical factor significantly associated with knowledge, attitude, and practice toward PCCS among nurses was the frequency of contact with PCCS. In addition, higher level of knowledge toward PCCS was associated with working in haematology and oncology wards, and participation in education related to palliative care. A more positive attitude toward PCCS was associated with working in a haematology and oncology ward, and experience of friends or relatives dying. Higher level of practice toward PCCS was associated with nurses who participated in education related to palliative care. In the path modeling, we found that holders of a master’s degree indirectly positive affected practice toward PCCS. Possession of a bachelor degree or above, being single, working within a haematology and oncology ward, and frequency of contact with PCCS positively affected practice toward PCCS.

**Conclusions:**

Based on this study, it is proposed that consultation with PCCS has a positive impact on the care of terminally ill patients. Encouragement of staff to undertake further education can improve the practice of ward staff providing palliative care.

## Background

Cancer is the leading cause of death in Taiwan. The number of deaths related to malignant tumors was 46,093, accounting for 28% of all deaths, in 2014 [1]. Because a large number of patients died from malignant tumours, it is important to improve the quality of life and ensure a dignified death of terminally ill patients. The Taiwanese government has advocated palliative care and enacted the Hospice Palliative Act since 2000 [2]. Palliative care not only serves patients but also assists their families to relieve physical, psychosocial and spiritual suffering [3].

The palliative care consultation service (PCCS) is another model of palliative care that has provided holistic end-of-life care for terminally ill patients in acute ward settings since 2005. The PCCS team is made up of the following members: doctors, nurses, psychologists, social workers, pharmacists, and spiritual care workers. They collaborated with primary health professionals to mainly provide advice, support, and guidance regarding palliative care [4, 5]. A study has confirmed the outcomes of PCCS, which revealed patients who received PCCS, received better symptom management, spiritual support and earlier development of resuscitation plans [6].

The percentage of patients receiving some form of palliative care support during the terminal phase of illness was less than 45% between 2006 and 2008 in Taiwan [7]. A qualitative study indicated that one of the leading barriers to referral of patients to receive palliative care was insufficient knowledge of palliative care by medical staff [8]. Several international studies also investigated the knowledge and attitude of nurses caring for end-stage cancer patients. Generally, these studies suggested that the knowledge and attitude of professionals in a palliative care team can indeed affect the evaluation of symptoms and subsequent treatment processes in terminally ill patients. In addition, the attitude of nurses was significantly associated with the probability of patients transferring to a palliative care facility [9, 10].

A literature review found that non-oncology nurses lacked education and training in cancer care and cancer treatment, which consequently impedes the delivery of quality nursing care for cancer patients [11]. Similarly, Coyne et al. found that the lack of knowledge for non-oncology registered nurses was detrimental to contact with PCCS, which was especially prominent in emotional support and communication with cancer patients and family members [12]. Nurses who lack knowledge about end-of life-care can negatively affect the decision making of terminally ill patients.

Previous studies have used multiple regression models to examine association between variables in the predictor of knowledge, attitude, and practice (KAP) and subsequent palliative care promotion [13, 14]. Although multiple regression models were crucial in applying information about the factors that predict KAP, we were interested in other factors that directly or indirectly impacted on practice toward PCCS. A prior study evaluated the direct or indirect factors affecting KAP regarding the use of restraints among nurses in rehabilitation settings through path analysis [15]. However, little is known about the relationship between KAP toward PCCS and utilizing path modeling in nursing staff. Therefore, the objective of this study was to explore the causal relationship between knowledge, attitude, and practice regarding PCCS using path modeling amongst Taiwanese nursing staff.

## Methods

### Study design and population

A descriptive cross-sectional research design was employed in a medical center in northern Taiwan in 2013, and this study was approved by the Institutional Review Board (IRB 2–102–05-128). A sample size of 255 was required to undertake regression analysis (using G* power 3.1). A two-tailed *p* value was set at 0.05, power at 0.9 and standardized regression coefficient at 0.2. We considered a 90% response and completion rate of the questionnaire; therefore, 285 subjects were recruited.

Convenience sampling was used to recruit nurses who fulfilled the inclusion criteria such as being a registered nurse, working in the ward or intensive care unit (ICU), able to communicate in Mandarin, and being willing and able to participate in this study. The researcher explained the objectives and methods to participants in the meeting room at ward meetings. Questionnaires were anonymous and information was confidential. Participants spent around 15–20 min to fill out the questionnaires. However, they were able to discontinue the questionnaire and withdraw from the study at any time. After completing the questionnaire, participants received a gift card. Only 1 subject refused to complete the questionnaire; therefore, data were collected from 284 nurses.

### Instruments

#### Demographics and work-related characteristics

Data collected included age, gender, education level, marital status, religious belief, length of service in nursing, ward, experience of death of friends or relatives, experience of death of patients while on duty, participation in education related to palliative care, and frequency of contact with PCCS.

#### Knowledge, attitude, and practice regarding PCCS inventory (KAP-PCCSI)

The KAP-PCCSI was developed based on the “self-reported questionnaire of nurses' knowledge and attitudes regarding end-of-life care [16]” and the “scale of palliative care knowledge and attitude” [17] as well as the literature regarding palliative care and information from experts. The KAP-PCCSI is composed of 3 scales measuring the knowledge (K-PCCSI), attitude (A-PCCSI), and practice (P-PCCSI) regarding PCCS of nurses.

The K-PCCSI was used to measure the level of understanding regarding the concept, purpose, significance, consultation process, and referral of patients. It contains 15 items; each item was rated on a 1–5 score with 1 being “no understanding” and 5 being “full understanding.” The total score ranges from 15 to 75, with a higher score indicating better knowledge in PCCS.

The A-PCCSI is composed of 10 items measuring attitudes toward PCCS team intervention and improving the quality of life of terminally ill patients. Items 9 and 10 were negatively worded. All items are rated on a 1–5 score with 1 being “strongly disagree,” and 5 being “strongly agree.” The total score ranges from 10 to 50, with a higher score indicating a more favorable attitude toward PCCS.

The P-PCCSI is composed of 10 items measuring the nurse’s ability to determine whether a patient meets the admission criteria, clearly explain and provide relevant information regarding PCCS to patients and relatives, discuss with the PCCS team regarding the patient’s condition or medical procedures, proactive learning of comfort care and terminal care techniques provided by the PCCS team, proactive communication with PCCS practitioners, and proactive sharing of personal thoughts. The items were rated on a 1–5 score with 1 being “never,” and 5 being “always.” The total score ranges from 10 to 50, with a higher score indicating more favorable practices regarding PCCS.

Content validity was established by a panel of 5 experts, which consisted of a professor, an assistant professor, a physician, a registered nurse and a social worker all working within the field of palliative care. Each item of KAP-PCCSI was rated on relevance, accuracy, and applicability on a 1–5 score by experts. The content validity index (CVI) for each item was counted the number of experts who rated the item on 4 or 5 score and divided that number by the total number of experts. The average CVI across the items in this study was 0.97. Cronbach’s alpha of the K-PCCSI, A-PCCSI, and P-PCCSI was 0.90, 0.86, and 0.93 for the 284 nurses, respectively.

### Statistical analysis

**Fig. 1:**
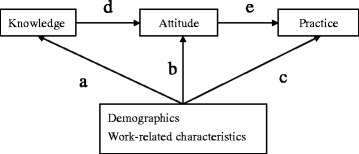
Path modeling

Statistical analyses were performed using statistical IBM SPSS software (SPSS 19 for windows). Continuous variables were descriptively expressed as means and standard deviations, and as frequencies and proportions for categorical variables. Data analysis included two parts. First, multiple linear regression analysis was used to examine the predictors for the KAP-PCCS of the nurses. Second, path analysis was used to describe the direct or indirect dependencies among a set of variables including demographics and work-related characteristics. Three types of results included demographics and work-related characteristics affecting practice through knowledge and attitude (a → d → e), affecting practice through attitude (b → e), and directly affecting practice (c). The path modeling is shown in Fig. 1. A *p* value of <0.05 was considered statistically significant.

## Results

### Demographics and work-related characteristics

The mean age of the participants was 29.1 years, and 69% were 25 years or older. Most of the subjects were female (95.8%), had a bachelor degree (74.6%), were single (79.2%), and had religious beliefs (62.0%). The mean length of service in nursing was 6.5 years, and 46.8% had a length of service of more than 5 years. The largest proportion of the participants worked in medical wards (38.7%), had experienced the death of friends or relatives (88.0%), and had experienced the death of patients while on duty (91.5%). A majority of the participants had participated in education related to palliative care (71.1%) and indicated having contact with PCCS 1–4 times (57.0%) (Table 1).

**Table 1:** Demographics and work-related characteristics (*N* = 284)

Variable	n	%
Demographics		
Age		
< 25 years	88	31.0
≥ 25 years	196	69.0
Mean ± SD	29.1 ± 6.4	
Gender		
Male	12	4.2
Female	272	95.8
Education level		
Junior college	59	20.8
Bachelor	212	74.6
Master	13	4.6
Marital status		
Single	225	79.2
Married	59	20.8
Religious belief		
No	108	38.0
Yes	176	62.0
Work-related characteristics		
Length of service in nursing		
< 1 year	67	23.6
1–5 year	84	29.6
> 5 year	133	46.8
Mean ± SD	6.5 ± 6.2	
Ward		
Hematology and oncology	28	9.9
Obstetrics and gynecology	17	6.0
Medical	110	38.7
Surgical	99	34.9
Intensive care unit	30	10.6
Experience of death of friends or relatives		
No	34	12.0
Yes	250	88.0
Experience of death of patients while on duty		
No	24	8.5
Yes	260	91.5
Participation in education related to palliative care		
No	82	28.9
Yes	202	71.1
Frequency of contact with PCCS		
0	31	10.9
1–4	162	57.0
≥ 5	91	32.0

*SD* standard deviation, *PCCS* palliative care consultation service

### Knowledge, attitude, and practice toward PCCS

Among the mean scores of knowledge, attitude, and practice, nurses scored the highest in attitude (mean score = 4.0) and lowest in practice (mean score = 3.6; Table 2).

**Table 2:** Knowledge, attitude, and practice toward palliative care consultation service among nurses (*N* = 284)

Variable	Range	Mean ± SD (total score)	Mean ± SD (item)	Q1,Q2,Q3
Knowledge	32–75	59 ± 8	3.9 ± 0.5	54, 59, 61
Attitude	24–50	40 ± 5	4.0 ± 0.5	36, 39, 43
Practice	11–50	36 ± 6	3.6 ± 0.6	33, 38, 40

*SD* standard deviation; *Q1* 25% quartiles; *Q2* 50% quartiles; *Q3* 75% quartiles

### Predictors of KAP-PCCS

As shown in Table 3, the significant predictors of the K-PCCS among nurses were ward, participation in education related to palliative care, and frequency of contact with PCCS, after adjustment for potential confounders. Nurses from medical wards, surgical wards, and ICU had lower mean scores for the K-PCCS than those from hematology and oncology wards by 7.2 points [95% CI (−10.2, −4.1), *p*<0.001], 5.0 points [95% CI (−8.1, −1.7), *p*<0.01], and 4.3 points [95% CI (−8.2, −0.3), *p*<0.05]. Nurses who have participated in education related to palliative care had a higher mean score for the K-PCCS than those who had never participated in relevant education by 2.0 points [95% CI (0.1, 3.9), *p*<0.05]. Compared with nurses who have never had contact with PCCS, those with 1–4 times and 5 or more times had a higher mean score for the K-PCCS by 4.5 points [95% CI (1.9, 7.1), *p*<0.001] and 8.2 points [95% CI (5.2, 11.2), *p*<0.001].

**Table 3:** Predictors for knowledge, attitude, and practice toward palliative care consultation service among nurses (*N* = 284)

	Knowledge		Attitude		Practice	
	Coefficients	(95% CI)	Coefficients	(95% CI)	Coefficients	(95% CI)
Constant	67.5		42.9		42.1	
Demographics						
Age						
< 25 years/≥25 years	0.1	(−2.5, 2.7)	0.4	(−1.5, 2.3)	−2.0	(−4.3, 0.4)
Gender						
Female/Male	3.7	(−0.3, 7.6)	0.9	(−2.0, 3.7)	0.8	(−2.8, 4.4)
Education level						
Bachelor/Junior college	0.1	(−1.8, 2.1)	0.2	(−1.2, 1.6)	0.1	(−1.7, 1.9)
Master/Junior college	3.3	(−0.8, 7.4)	2.5	(−0.5, 5.5)	1.9	(−1.9, 5.6)
Marital Status						
Married/Single	1.7	(−0.5, 4.0)	−0.3	(−1.9, 1.3)	1.2	(−0.8, 3.2)
Religious Belief						
Yes/No	1.1	(−0.5, 2.7)	0.7	(−0.4, 1.9)	1.2	(−0.3, 2.7)
Work-related characteristics						
Length of service in nursing						
1–5 years/<1 year	−1.1	(−3.9, 1.7)	0.1	(−1.9, 2.1)	−0.1	(−2.6, 2.5)
> 5 years/<1 year	−1.7	(−4.9, 1.6)	−0.3	(−2.7, 2.0)	−0.3	(−3.2, 2.7)
Ward						
Obs-Gyn/HemaOnco	−0.4	(−4.6, 3.8)	−3.5	(−6.5, −0.4)*	2.4	(−1.4, 6.2)
Medical/HemaOnco	−7.2	(−10.2, −4.1)***	−4.1	(−6.3, −1.9)***	−2.2	(−5.0, 0.6)
Surgical/HemaOnco	−5.0	(−8.1, −1.7)**	−3.6	(−5.9, −1.3)**	−0.3	(−3.2, 2.6)
ICU/HemaOnco	−4.3	(−8.2, −0.3)*	−4.5	(−7.3, −1.6)**	−0.5	(−4.1, 3.0)
Experience of death of friends or relatives						
Yes/No	0.7	(−1.8, 3.2)	1.9	(0.1, 3.7)*	0.01	(−2.3, 2.3)
Experience of death of patients while on duty						
Yes/No	1.1	(−1.9, 4.2)	0.4	(−1.9, 2.6)	0.9	(−1.8, 3.7)
Participation in education related to palliative care						
Yes/No	2.0	(0.1, 3.9)*	0.6	(−0.7, 2.0)	1.7	(−0.04, 3.4)*
Frequency of contact with PCCS						
1–4times/0	4.5	(1.9, 7.1)***	1.9	(0, 3.8)*	3.1	(0.7, 5.4)
≥ 5times/0	8.2	(5.2, 11.2)***	3.2	(1.0, 5.4)**	6.0	(3.3, 8.7)***

*P<0.05, **P < 0.01, ***P < 0.001

*ObsGyn* obstetrics and gynecology, *HemaOnco* hematology and oncology, *ICU* intensive care unit, *PCCS* palliative care consultation service, *CI* confidence interval

Ward, experience of the death of friends or relatives, and frequency of contact with PCCS were important predictors for the A-PCCS after adjustment for potential confounders. Nurses in the obstetrics and gynecology wards, medical wards, surgical wards, and ICU had a lower mean score in the A-PCCS than those in the hematology and oncology wards by 3.5 points [95% CI (−6.5, −0.4), *p*<0.05], 4.1 points [95% CI (−6.3, −1.9), *p*<0.001], 3.6 points [95% CI (−5.9, −1.3, *p*<0.01], and 4.5 points [95% CI (−7.3, −1.6), *p*<0.01]. Nurses who have experienced the death of friends of family had higher mean scores than those who have not by 1.9 points [95% CI (0.1, 3.7), *p*<0.05]. Compared with the nurses who have never had contact with PCCS, those with 1–4 times and 5 or more times showed an increased mean score for the A-PCCS by 1.9 points [95% CI (0, 3.8), *p*<0.05] and 3.2 points [95% CI (1.0, 5.4), *p*<0.01].

Participation in education related to palliative care and frequency of contact with PCCS were significant predictors of the P-PCCS among nurses. Nurses who have participated in education related to palliative care had a higher mean score for the P-PCCS than those who have never participated in relevant education by 1.7 points [95% CI (−0.04, 3.4), *p*<0.05]. Compared with the nurses who have never had contact with PCCS, those with 5 or more times had a higher mean score for the P-PCCS by 6.0 points [95% CI (3.3, 8.7), *p*<0.001].

### Path modeling of knowledge, attitude, and practice

The path modeling demonstrated that having a master’s degree significantly affected practice through knowledge and attitude compared to colleagues with an undergraduate degree (Coefficients = 0.169, *p* = 0.004). The model also showed that educational level, marital status, ward, and the frequency of contact with PCCS directly affected practice. The relationships between bachelor/junior college (Coefficients = 0.176, *p* = 0.003), master’s/junior college (Coefficients = 0.376, *p* < 0.001) and practice were significant by standardized coefficient estimates for the paths. Being married was significantly negative in relation to practice compared to being single (Coefficients = −0.124, *p* = 0.037). Working in a medical ward (Coefficients = −0.324, *p* < 0.001), surgical ward (Coefficients = −0.232, *p* < 0.001), or ICU ward (Coefficients = −0.293, *p* < 0.001) were also significantly negative in relation to practice compared to working in a hematology or oncology ward. In addition, comparison of nurses who had never contacted PCCS, with those who had contacted PCCS for 1–4 times showed a positive significant correlation with practice (Coefficients = 0.220, *p* < 0.001). The above data are shown in Table 4.

**Table 4:** Path modeling for knowledge, attitude, and practice toward palliative care consultation service among nurses (*N* = 284)

	Knowledge → Attitude → Practice		Attitude → Practice		Practice	
Variables	Coefficients	*P* value	Coefficients	*P* value	Coefficients	*P* value
Demographics						
Age						
< 25 years/≥25 years	−0.015	0.797	0.006	0.926	−0.019	0.750
Gender						
Female/Male	0.002	0.974	−0.049	0.413	−0.012	0.840
Education level						
Bachelor/Junior college	−0.003	0.956	0.009	0.880	0.176	0.003
Master/Junior college	0.169	0.004	−0.064	0.284	0.376	<0.001
Marital Status						
Married/Single	−0.034	0.570	0.010	0.866	−0.124	0.037
Religious Belief						
Yes/No	0.016	0.792	−0.009	0.885	−0.091	0.126
Work-related characteristics						
Length of service in nursing						
1–5 years/<1 year	0.001	0.988	0.010	0.868	−0.005	0.933
> 5 years/<1 year	0.020	0.734	0.006	0.915	0.052	0.383
Ward						
ObsGyn/HemaOnco	−0.078	0.191	0.048	0.418	0.005	0.933
Medical/HemaOnco	0.001	0.993	0.001	0.985	−0.324	<0.001
Surgical/HemaOnco	−0.023	0.703	0.014	0.818	−0.232	<0.001
ICU/HemaOnco	−0.089	0.133	0.070	0.238	−0.293	<0.001
Experience of death of friends or relatives						
Yes/No	−0.016	0.791	−0.048	0.422	0.044	0.460
Experience of death of patients while on duty						
Yes/No	0.0003	0.996	0.001	0.993	−0.085	0.153
Participation in education related to palliative care						
Yes/No	−0.061	0.310	−0.028	0.639	−0.055	0.356
Frequency of contact with PCCS						
1–4times/0	0.013	0.833	−0.016	0.786	0.220	<0.001
≥ 5times/0	0.033	0.575	−0.024	0.687	0.479	0.750

*ObsGyn* obstetrics and gynecology, *HemaOnco* hematology and oncology, *ICU* intensive care unit, *PCCS* palliative care consultation service

## Discussion

### Factors affecting the KAP-PCCS among nurses

This study found that working within a haematology and oncology ward, participation in education related to palliative care and frequency of contact with PCCS were positive predictors of knowledge towards PCCS. Most recent studies suggest that participation in palliative care education programs, years of nursing experience, working in an oncology ward, having a close family member or friend who had used palliative care services, and palliative care integration into the health-care system of the country were positively associated with knowledge of palliative care among nurses [14, 18–20]. Regarding the knowledge aspect of their practice, the majority of nurses reported that recognition of palliative care needs often occurred at the terminal stage, and then was carefully taken into consideration while dealing with the spiritual and medical needs of the patient [13]. Nurses who had received training in palliative care and perceived themselves to be knowledgeable in palliative care demonstrated increased awareness of palliative care and could improve the quality of care in terminally ill patients [21, 22]. Therefore, it is crucial to assess the need for terminal care education in nurse training programs and provide continuing end-of-life nursing care education to strengthen non-specialist wards in end-of-life care knowledge.

This study found that working in a haematology and oncology ward, experience of a family member or friend who had died, and frequency of contact with PCCS were significant positive predictors of the attitude of staff towards PCCS. Several studies have demonstrated groups of staff who have a more favourable attitude toward palliative care. This includes: 1) age – older; 2) marital status - being married; 3) education - having a higher degree; 4) longer time working in an oncology or medical ward; 5) participation in palliative care training courses; 6) had worked in nursing for a longer duration; 7) experience in caring for patients and their families at end-of-life. Integration of palliative care into the health-care system also influenced attitudes towards palliative care of staff [14, 18, 20, 23–25]. Therefore, nurses’ knowledge and attitude toward palliative care have critical implications on the quality of care for terminally ill patients. Lack of knowledge and skill among nurses prevent effective care for end-stage cancer patients. Based on this evidence, it can be inferred that receiving education in palliative care or palliative related training programs reinforces a more positive attitude toward palliative care.

Nurses’ involvement in proactive palliative care is important in supporting patients’ palliative care management. As indicated by the results of this study, participation in education related to palliative care and frequency of contact with PCCS are important predictors affecting nursing practice regarding PCCS. White and Coyne examined the knowledge, attitude, and practice regarding end-of-life care among 714 oncology nurses and indicated that continuing education regarding palliative care was important for improving their clinical practice [26]. Therefore, we recommend incorporating palliative care into the nursing training program to increase knowledge and improve attitude and practice regarding PCCS amongst nurses. This will help ensure that end-stage patients receive more comprehensive palliative care, ultimately improving the quality of life of terminally ill patients.

### Path modeling of knowledge, attitude, and practice

Path analysis is a method for examining causal patterns among a set of variables [27]. The results of our path analysis indicated that bachelor degree or above, being single, working in a haematology and oncology ward, and frequency of contact with PCCS had positive direct effects on liaison with PCCS. Having a master’s degree had a positive, although indirect effect, on nurse practice through the influence on knowledge and attitude. This implies that nursing staff with a higher educational level would have more knowledge about PCCS, which indirectly resulted in more favorable attitude and better practice toward PCCS. A previous study indicated that knowledge of palliative care was associated with attitude and coping with the death of terminally ill patients, while attitude was related to coping practices. Nurses’ attitudes and coping skills were related to preparedness to practice in palliative care [13]. According to the KAP theory, practice is an individual’s response to stimulation. It is an actual presentation that is closely related to an individual’s knowledge and attitudes [28]. As lack of knowledge has some influence on nursing staff attitude and practice toward PCCS, we suggest that education is especially important in changing these attitudes and practices.

Being single was associated with positive direct effects on practice towards PCCS in this study. A prior study indicated that single nurses had better attitudes toward palliative care than married nurses in south-east Iran [23]. However, this result was different from another similar study amongst Korean nurses [24]. Nurses working in a haematology and oncology ward were influenced in their practice more by PCCS than those work worked in medical and surgical wards and the ICU. It is likely that nurses working in a haematology and oncology ward had more opportunities to experience how to take care of terminal patients in their daily practice, which may help them to refer patients to PCCS specialists. The frequency of contact with the PCCS also positively influenced practice and interaction with the PCCS. In our clinical practice, the primary nurse who contacted the PCCS team members could discuss the terminal patients’ condition with them directly. Non-specialist ward nurses reported a lack of training in relation to cancer care which could impede the ability to provide high quality nursing care to patients with cancer diagnoses. In addition, emotional support and communication ability with patients and their families could also distress non-specialist nurses. Therefore, it is important that the practice of contacting PCCS team members to learn and develop clinical ability is encouraged. This would assist in providing supportive and holistic care for both patients and their families.

### Limitations and recommendations

This study has a number of limitations. First, the KAP-PCCSI was a nurse-reported assessment tool. Because of differences between Chinese and Western cultures, Taiwanese nursing staff may be reluctant to report their true opinions because of concern about being different to colleagues. As a result, KAP-PCCSI might be over-reported by nurses and the true referral rate of PCCS was unknown. Second, as our study population was a convenience sample and limited to one medical center, the generalizability of our findings is limited. Third, this study used a cross-sectional design, meaning that change in KAP-PCCS over time was not investigated. Finally, nursing staff working in a haematology and oncology ward know how to treat patients with oncology diagnoses, have basic concepts of palliative care and receive the best training programs in order to become a haematology and oncology nurse. However, it does not necessarily mean these programs will assist them to provide the best palliative care for patients, particularly for those diagnosed with a non-cancer illness.

Based on the results of this study, future research directions are indicated. First, studies using additional empirical assessment tools or calculating referral rate of PCCS are needed to determine the KAP-PCCS level. Second, random sampling and large sample research in Taiwan is needed to confirm our findings. Third, studies using a longitudinal design are needed to explore the changes in KAP-PCCS among nursing staff. Finally, extra training in palliative care and access to PPCS is integral to nurses providing the best palliative care for patients.

## Conclusions

This study suggest that working within a haematology and oncology ward, participation in education related to palliative care, and frequency of contact with PCCS were significantly predictors of knowledge toward PCCS. Working in an haematology and oncology ward, experience of death of friends or family, and frequency of contact with PCCS were significantly predictors of attitude towards PCCS. Finally, participation in education related to palliative care and frequency of contact with a PCCS were the key predictors in relation towards practice and interaction with a PCCS. In addition, holding a master’s degree indirectly affected practice through knowledge and attitude. Holding a bachelor degree or above, being single, working within a haematology and oncology ward, and frequency of contact with PCCS directly influenced practice regarding palliative care. Based on the findings of this study, we suggest nurses should have access to a PCCS team for consultation and the opportunity to discuss patients with requiring palliative care. This may allow nurses to appreciate the importance of palliative care, improve palliative nursing practices, and enhance the quality of care for patients receiving end-of-life-care.

## Abbreviations

A-PCCSI: Attitude regarding PCCS inventory
CI: Confidence interval
CVI: Content validity index
HemaOnco: Hematology and oncology
ICU: Intensive care unit
KAP: Knowledge, attitude, and practice
KAP-PCCSI: Knowledge, attitude, and practice regarding PCCS inventory
K-PCCSI: Knowledge regarding PCCS inventory
ObsGyn: Obstetrics and gynecology
PCCS: Palliative care consultation service
P-PCCSI: Practice regarding PCCS inventory
Q1: 25% quartiles
Q2: 50% quartiles
Q3: 75% quartiles
SD: Standard deviation
